# How did you perform? Investigating football players’ perception of self-regulated passing performances under auditory noise environments

**DOI:** 10.3389/fpsyg.2024.1390487

**Published:** 2024-04-29

**Authors:** Stefanie Klatt, Fabian Werner Otte, Adam Beavan, Tom Schumacher, Sarah Kate Millar

**Affiliations:** ^1^Section Cognition in Team Sports, Institute of Exercise Training and Sport Informatics, German Sport University Cologne, Cologne, Germany; ^2^TSG Research Lab gGmbH, Zuzenhausen, Germany; ^3^Faculty of Health, School of Health Sciences, University of Canterbury, Christchurch, New Zealand

**Keywords:** affective learning design, representative learning design, athlete self-regulation, Ecological Dynamics, Nonlinear Pedagogy, Footbonaut, skill learning, auditory cues

## Abstract

**Introduction:**

This paper deals with the question on how sport performances may be influenced by internal, emotional processes, which stem from outside feedback.

**Methods:**

In terms of methods, players’ subjective performance ratings for four experimental auditory cue conditions were examined; these included both ‘positive’ and ‘negative’ stadium noise, ‘no (auditory) conditions,’ and a control/‘baseline’ condition. This resulted in a qualitative-analytic data set that was obtained succeeding each auditory cue condition using a unique football training machine (i.e., known as ‘Footbonaut’). Without having received any coaching/performance feedback, players were asked to rate and individually comment on their perceived performance ratings for each experimental auditory condition.

**Results:**

Findings indicate stronger and more significant correlations between auditory conditions and subjective ratings compared to the non-auditory condition and its subjective rating. Furthermore, data provides initial insight into players’ emotional experiences during each of the practice conditions.

**Discussion:**

These noteworthy findings on players’ abilities to accurately judge their performances based on selfmonitoring and intrinsic feedback are discussed from an Ecological Dynamics perspective, linked to a Nonlinear Pedagogy for coaching. Here, representative and affective learning designs for skill learning and performance preparation are presented. Finally, a hypothetical catalyst effect of auditory stadium noise on subjective performance rating is proposed.

## Highlights

- This study aims to better understand players’ emotional experiences of auditory noise environments and their subjective abilities to accurately perceive and judge their performances to them.
- Results show that skilled football players use self-monitoring and intrinsic feedback to judge their performances; and emotionally and positively respond to (game-representative) auditory noise environments.
- A link between auditory information, its effects as a catalyst on task performance and subjective emotional regulation is proposed.
- Findings underline the benefits of representative and affective learning designs and a hands-off coaching approach.

## Introduction

During the COVID-19 pandemic, professional sports leagues worldwide experienced significant disruptions, often halting mid-season. Association football, with the first national league to resume being the German Bundesliga, tried to restart its schedule under stringent regulation. Notably, stadiums remained devoid of spectators—a measure intended to mitigate the spread of the virus. Consequently, professional players encountered a peculiar situation: the once vibrant and emotionally charged atmosphere of stadiums was replaced by silence. This prompts an intriguing inquiry: Did this change in auditory information affect the players temporarily in their playing ability, and if so, how?

Research into stadium noise and its impact on performance has surged during the COVID-19 pandemic. A meta-analysis by Leitner et al. (2023) comprehensively examines numerous studies conducted during the pandemic era, focusing on the home-field advantage. While the home-field advantage has been well documented across various sports and contexts by scientists over the last 30 years, no one clearly dominant factor for it has been established (Legaz-Arrese et al., 2012). Rather, research highlights a multitude of causes, such as crowd and travel effects, territoriality, referee bias, and other psychological factors (see Pollard, 2008, for an initial review). Here, Pollard (2008, p. 13) stresses that “ultimately it is what goes on in the mind of players, coaches and referees that determine their actions and hence the result of a game and the role played by home advantage.” Connecting this research area on home-field advantages along with potential crowd noise effects back to the current study, it is of interest to what degree emotionally-laden (positively or negatively perceived) auditory stadium environments – in contrast to silent, no noise (COVID-19 pandemic-like) environments – may impact player performances, perceptions, enjoyment, and motivation (Otte et al., 2021). While existing studies predominantly rely on in-field analytics, investigations into athletes’ behavior amidst altered auditory environments remain scarce. Previous studies examining the influence of stadium noise under controlled laboratory settings, such as Otte et al. (2021), have primarily focused on objective metrics like passing accuracy and time. These experiments revealed that athletes exhibited quicker passing times when exposed to pertinent auditory cues compared to negative (e.g., booing) or silent conditions. However, these findings, though valuable, present an incomplete picture. The fundamental question that remains unanswered is the underlying mechanism driving divergent behavioral responses across varied noise conditions.

This paper aims to address this research gap by delving into the subjective experiences and perceptions of athletes amidst varying stadium noise levels. By analyzing a previously collected yet unexplored qualitative dataset concerning football players’ perceived performances under different auditory cue conditions, we aim to shed light on the nuanced interplay between auditory stimuli and athletes’ cognitive and emotional responses. This novel approach will not only enhance our understanding of athletes’ adaptability to stadium noise but also elucidate their ability to evaluate performance independently of coach-led feedback. Furthermore, we seek to correlate these perceptual abilities with players’ emotional engagement with distinct football-specific auditory cues.

## Key concepts in Ecological Dynamics to highlight the importance of emotional processing in regards to self-regulation and self-monitoring

To understand why this research is a welcoming contribution to the expanding literature on behaviors of football players in different auditory contexts, we first must explain why it is important to view this study from the lens of the athlete’s emotional perspective. To do so, we will use an Ecological Dynamics perspective, highlighting the deeply intertwined relationships between perception, action, cognition, intentions, and emotions. From this perspective, understanding athletes as complex and adaptive systems, composed of numerous interacting parts, is critical (Phillips et al., 2010). Particularly, it is the scale of analyzing athletes’ performances holistically on perceptual-cognitive, physical and emotional levels that further concerns their ability to self-regulate under varying contexts (Davids, 2015). For practice and competition contexts, it is therefore the coach’s role to guide players’ *self-regulation* and *self-monitoring* toward goal-oriented and functional behavior (Davids, 2015; Woods et al., 2020a). Self-regulation is understood as the human capacity to manage ones urges according to previously defined goals or ideas (Baumeister et al., 2007). These goals/ideas can be both from an external source but also stem from an internal one. An important subcomponent of self-regulation is self-monitoring, and as laid out by Zimmerman (2000, 2001), self-monitoring displays a way for the individual to implicitly sense and assess whether the current task is done effectively from the person’s own point of view. More importantly, papers from Diamond and Aspinwall (2003) or Bridgett et al. (2013) showcase that emotions can heavily affect the self-regulation process. These authors state that while negatively charged emotions often hinder the transfer of mental into task-related skills, positively charged emotions facilitate this transfer. If we keep the previous definition in the back of our mind, it becomes therefore essential to analyze the player’s own perceived emotional state, as without it, our ecological view would miss a key variable.

In addition, the bidirectionality of the *player-environment relationship* provides some clear principles for guiding the design of practice activities (Woods et al., 2020a). For example, the use of nonlinear pedagogical concepts, such as *representative learning* and *affective learning designs*, has been advocated by research for numerous years (see Otte et al., 2019, 2020, and Headrick et al., 2015, for recent conceptual discussions for each learning design, respectively). Representative learning designs emphasize the notion for practice activities to replicate constraints and key information that is present in the competitive performance environment (Woods et al., 2021), whereas affective learning designs highlight the embedment of emotions into these representative (practice) tasks, potentially evoking individualized behavioral tendencies in different athletes (Headrick et al., 2015). These constraints are defined as part of the Constraints-Led Approach, which is in turn underpinned by principles of Ecological Dynamics and Nonlinear Pedagogy (Renshaw et al., 2016; Button et al., 2020). Constraints are viewed as individual, task-related and environmental characteristics and features that guide a player’s search for and perception of relevant information. Examples can be objects, like specific passing targets or auditory conditions, such as stadium noise (e.g., Fajen et al., 2009). From an applied coaching perspective, it would therefore be ideal if one uses constraint manipulations (e.g., adding stadium noise conditions) to design these representative and affective practice environments, which focus on holistically integrating all performance-regulating sub-systems (i.e., perception, action, cognitions, intentions, and emotion; Woods et al., 2020a). Put simply, the practice design and its constraints drive athlete self-regulation and exploration (Woods et al., 2020b). These processes are intentionally regulated in constant interaction between athletes and their surrounding environments (Davids et al., 2015).

Without further coach-induced or similar types of augmented feedback, athletes learn to search for and perceptually attune to relevant environmental information and invitations for action (also termed affordances; see Fajen et al., 2009). It is important to note that search in this case stems from the perceptual-cognitive entanglement, highlighting how externally and internally perceived information are mutually dependent in driving athlete self-regulation. An example of this would be an internal appraisal of a whistling crowd, which would startle the athlete. This in an essence means that in absence of augmented feedback, people (and athletes) aim to enhance the use of intrinsic (sensory) feedback sources such as emotional feedback to self-monitor and adapt task-specific behavior (Hodges and Franks, 2002; Otte et al., 2019, 2020). For example, a football player would always feel and see the consequence of a pass without receiving further extrinsic and augmented coaching feedback (Williams and Hodges, 2005). Particularly, the notions of task-intrinsic feedback and self-monitoring relate to this investigation, in that it aims to examine skilled football players’ abilities to accurately, and independently, judge their own performances. This idea may be further supported by previous research demonstrating athletes’ abilities to use acquired and specified knowledge to accurately assess movement performances (Hadfield, 2005; Fajen et al., 2011; Millar et al., 2011, 2017). For example, Millar et al. (2017) found Olympic rowers show accuracy in judging and successfully identifying quality rowing stroke performances by accessing knowledge of their performances. This finding may be extended by research demonstrating expert rowers to accurately perceive and monitor their own catch efficiency, which was objectively reflected by changes in boat speed (Millar et al., 2017). While to the authors’ knowledge, there has been no investigation to date into football players’ abilities to accurately self-monitor performances (under varying noise conditions), previous research commonly emphasizes “high-level performers as expert systems adept at detecting and evaluating change focussed on performance” (Millar et al., 2015, p. 3). Yet a special importance of these ecological factors and emotions so far has neither taken place in research, nor in the common training regimen of athletes. In an essence, this leads the training tasks to become non-representative or at least less representative for a stadium-based (noise) atmosphere. This could lead to a missing transfer of training skills in a professional sporting environment, these being the stadium environment. Consequently, based on the proposed theoretical rationale with a focus on emerging player-environment relationships (Davids et al., 2008) and the existing research gap, this article aims to investigate:

- To what extent skilled football players accurately judge their performance in absence of augmented, coach-led performance feedback; and
- How football players perceive and self-regulate their emotional reactions to various auditory cue environments in practice.

## Method

### Participants

An initial *a priori* analysis was conducted to determine the required sample size for this study using the computer program G*power 3.1.9.7. The estimated effect size for this study was unknown, as the few studies that analyzed stadium noise at a professional level all failed to include effect sizes. However, conceptually similar studies focusing on auditory stimuli affecting treadmill walking (*η*^2^ = 0.24; Karageorghis et al., 2009) and running performance (*η*^2^ *=* 0.20; Bood et al., 2013) demonstrated rather large effects. We, therefore, estimated the participants of a correlation analysis using a medium to strong effect of *r* = 0.6 with a relative power of 0.80 and a critical alpha of 0.05. This resulted in 19 participants that we needed to recruit for this study. Unfortunately, this margin was missed by four participants due to the requirement of a highly specialized sample size of elite athletes. Therefore, the final participant number of the experiment is a sample of 15 male football players (*n* = 15, U23s age group) and results from this study should be taken with care due to possible type-1 error inflation. The ethical approval for the presented study protocols was granted by the lead author’s university ethics commission in 2019.

### Procedure

The highly skilled sub-elite players were tested on objective metrics such as their passing performances [i.e., passing accuracy score (in %) and average passing time (in s)], using the standardized and validated robotic football training tool, known as ‘Footbonaut’ (CGoal GmbH, Berlin, Germany; see Beavan et al., 2019, and McGowan, 2012). In said training, after a warmup procedure consisting of 10 passing repetitions, the players were instructed to perform four identical football passing rounds consisting of 32 low passes over the course of 2–3 min. The Footbonaut is a high-tech robotic cage where footballers can improve their technical skills without any other players (Beavan et al., 2019). The four sessions differed due to different randomized auditory noise conditions. These four different conditions are: (1) A ‘Baseline’ condition: the training environment allows for participants to perceive all relevant visual information (i.e., light signals) and auditory cues (i.e., ‘beep’ sound signals at a volume of approximately 75 dB) on passing source and passing target ‘window,’ as provided by the Footbonaut (i.e., participant’s hearing was not distracted). (2) A ‘No auditory’ cue condition: the training environment significantly limits the participant’s perception of auditory information (i.e., ‘beep’ sound signals) on passing source and target ‘window’ provided by the Footbonaut (i.e., participants were asked to wear ear defenders throughout the training session). (3) A positive auditory cue condition: the training environment displays loud stadium noises (i.e., a football crowd singing) played through speakers in the Footbonaut (i.e., with a volume of approximately 85 dB); thus, the participant’s perception of auditory information (i.e., ‘beep’ sound signals) on passing source and target ‘window’ provided by the Footbonaut are impaired. or (4) A negative auditory cue condition: the training environment displays loud stadium noises (i.e., a football crowd whistling and ‘booing’) played through speakers in the Footbonaut (i.e., with a volume of approximately 85 dB); thus, the participant’s perception of auditory information (i.e., ‘beep’ sound signals) on passing source and target ‘window’ provided by the Footbonaut are impaired. The crowd sounds of fans singing and chanting were pre-tested for their validity (Otte et al., 2021).

The task was instructed to the players at the beginning, indicating that they should “receive and pass the ball as quickly as possible.” All conditions were completed by each participant in a randomized order. Notably, emotional valence of auditory stadium conditions was pre-tested by 30 participants (*n* = 30) and the Footbonaut training machine allowed the researchers to control for various variables (e.g., passing repetition numbers, ball release speeds and angles from the machine, light and ball conditions). Additionally, all available information remained the same for each passing repetition per session (e.g., visual information, auditory conditions, ball speed and trajectories; see Otte et al., 2021).

After all the information from the participants was recorded, feedback was provided for the athlete by the lead experimenter and the participant debriefed and dismissed.

### Measures

Additionally, to the physical data already analyzed by Otte et al. (2021) with help of the Footbonaut, players were also asked to provide subjective statements regarding their own performance. These subjective statements will be the focus for the following analysis. Without disclosing players’ performance scores nor providing any augmented (verbal) coaching feedback, players were asked to provide both standardized and open statements following each of the four auditory conditions (i.e., under the ‘baseline,’ ‘no auditory cue,’ ‘positive auditory cue’ and ‘negative auditory cue’ conditions). Recordings were made by athletes answering a questionnaire after completing each practice condition. The lead experimenter was always present during the data collection of the Footbonaut and handed out the questionnaire, however the experimenter was not present during the time the athlete was filling out the answers. To further control for possible cognitive and emotional biases during the experiment, there was no feedback or consultations provided regarding the information on the questionnaires for the athletes during the four conditions. In detail, ratings and perceptions after each auditory cue conditions were measured in two ways. Both of these measurements were collected in a previous study (Otte et al., 2021), but were not analyzed upon:

1. Players were asked to rate their performance for each session on a Likert-type scale from 0 (i.e., strongly unsatisfied) to 10 (strongly satisfied); and
2. Players were questioned on their performances and subjective perceptions of the auditory cue conditions. In particular, they were asked to ‘please comment on your perceptions of/ feelings about each auditory cue conditions’ after each of the four sessions.

### Data analysis

For the data analysis, subjective ratings and statements were compared to objective performance data obtained from the Footbonaut system, as previously mentioned in Otte et al. (2021), but briefly summarized here. The results of this experiment revealed that under negatively valenced sounds, such as negative auditory conditions or absolute silence, reaction times in the Footbonaut were slower. Conversely, positively valenced sounds, such as cheerful crowd noise, did not yield any significant improvements. Similarly, passing accuracy was not affected by any of the auditory conditions. To establish a connection with the present study, a Spearman-rho correlation analysis was employed to investigate whether subjective emotional scores are associated with average reaction time and passing accuracy. This correlation analysis was conducted for each of the eight conditions, and results are presented in Table 1.

**Table 1:** Overview of the different correlations between the different subjective emotional categories and the objective stats.

	Accuracy score	Average time taken
Baseline	0.657*	0.413
Positive	0.841*	0.102
Negative	0.790*	−0.131
Noise-canceling headphones	0.514	0.246

Significant correlations (*p* < 0.05) are marked with an asterisk (*).

Additionally, a manipulation check was done to see whether participants correctly can assess their subjective performance this was done by comparing the perceived subjective rating to the accuracy achieved in the Footbonaut.

Qualitative-analytic exploration considered each player’s standardized ratings and subjective statements following each auditory cue conditions. Here, players’ perceived performance ratings (i.e., between 0 and 10) were used for correlation analysis with the two objective performance scores (i.e., passing accuracy and average passing time). Further, an inductive, data-driven theming process was used to code the qualitative, open statements that players provided after each auditory cue conditions (Braun and Clarke, 2006). Specifically, in order to explore players’ subjective statements regarding each auditory cue condition, thematic analysis allowed to examine individual players’ perspectives and feelings (King, 2004; Nowell et al., 2017).

A thematic analysis was conducted by the experimenters based on the open statements voluntarily filled out by each player. The result from these statements were filtered and subsequently divided into three coding themes, which include: (i) ability to perceive acoustic information; (ii) perceived positive influence on performance; and (iii) perceived positive influence on emotional engagement. All experimenters spoke the native language of the athletes, German.

## Results

Qualitative-quantitative exploration of players’ subjective statements following each Footbonaut auditory cue conditions provides insight into: (1) the correlation between players’ subjective performance ratings and two objective performance data measures of passing accuracy and average passing time; and (2) players’ subjective and internal emotional regulations to the auditory cue conditions.

### Players’ subjective performance ratings

A spearman-rho correlation analysis was performed with all of the different emotional conditions (baseline, positive, negative, and headphones) in relation to average passing time and passing accuracy. Due to time-related issues, one participant out of 15 only managed to be tested in the objective conditions and could not be questioned regarding their subjective emotional analysis. This participant was excluded in the correlational analysis. In the baseline subjective rating condition, we saw a correlation of 0.657 (*p* = 0.011) for the baseline accuracy and a correlation of 0.413 (*p* = 0.142) for the average time taken. The positive subjective rating condition was correlated to the accuracy with 0.841 (*p* < 0.001) and not correlated to the average time with 0.102 (*p* = 0.729). The negative subjective rating condition was correlated to the accuracy with 0.790 (*p* = 0.001) and not correlated to the average time with −0.131 (*p* = 0.657). The headphone subjective rating condition was not correlated to the accuracy with 0.514 (*p* = 0.06) nor to the average time with 0.246 (*p* = 0.397) for the average time taken. Table 2 presents an overview of all correlations. Significant correlations are marked with an asterisk (*).

**Table 2:** Spearman-rho correlational analysis between subjective performance ratings and their accuracy performance score, where a simple yes (√) or no (X) represents when each player was accurate about their highest (or lowest) subjective rating, matches their highest (or lowest) accuracy score or time taken.

Player	Highest score	Fastest time	Lowest score	Slowest time
1	√	√	X	√
2	√	X	√	X
3	√	X	√	√
4	√	√	√	X
5	√	X	√	X
6	√	√	√	√
7	√	√	√	X
8	√	√	X	√
9	√	√	√	X
10	√	√	√	√
11	√	X	√	X
12	√	X	√	X
13	X	X	X	√
14	√	X	√	X
Averages	93%	50%	79%	43%

Furthermore, as a validation check, Table 2 presents a qualitative analysis of a simple yes (√) or no (X) to represent when each player was accurate about their highest (or lowest) subjective rating, matches their highest (or lowest) accuracy score or time taken. For example, a tick (√) indicated a direct match between subjective rating and a performance score. Here, it can be observed in Table 1 (highest score), that 93% of players were accurate and knew when they had their most accurate round (of the four conditions) and subjectively rated it their highest. Likewise, players were mostly accurate to know when they had their lowest score. While players were less accurate about their performance time, all 14 players were accurate about at least one of the four measures and over 55% of players were accurate in 3 of the 4 measures. When all measures were taken together (*n* = 56), a significant correlation of 0.735 (*p* < 0.001) was given between own subjective rating and performance of the players. A non-significant correlation of 0.132 (*p* = 0.13) was shown between subjective rating and time taken of the scores.

### Players’ subjective perceptions of the auditory cue conditions

Qualitative analysis of players’ subjective perceptions and statements after each practice round led to three coding themes; these include: (i) ability to perceive acoustic information; (ii) perceived positive influence on performance; and (iii) perceived positive influence on emotional engagement. Notably, due the analysis of participants’ perceptions of each practice condition concerned open and voluntary statements (i.e., statements about the ‘baseline,’ ‘no auditory cue,’ ‘positive auditory cue,’ and ‘negative auditory cue’ conditions). First, the ‘baseline’ practice condition was clearly perceived to be *“easiest”* in regard to multimodal cues providing support for passing performance. Its accessibility concerning perceiving acoustic information to support their passing performances was stated by 10 of 14 players (*n =* 10). For instance, while one player stated that “*signals are clearly hearable*,” another player expressed that “*one can perceive both noises (ball machine and target) very well and one can often find the optimal position*.” In regard to this condition’s impact on emotional engagement, remarkably no player made a statement leading to the notion that the baseline condition had a rather neutral effect on emotional engagement.

Second, eight players (*n* = 8) claimed that the ‘noise-canceling headphones condition’, as expected, had influence on their performances by, for example, making statements such as: headphones made it “*difficult to hear the signals*” and (more) “*difficult to perceive the noises*.” Whereas these statements indicate perception of increased practice task complexity under a ‘no auditory cue condition,’ mixed feelings regarding its influence on performance were specified (i.e., eleven players mentioned for this condition to either have positive or negative impact on performance). For example, some players provide statements such as: *“No noise helps because I was more concentrated through the silence”* and “*I felt more concentrated and force[d] to find more [visual] orientation*.” In contrast, other players mentioned this cue condition to be “*harder*,” because “*too much concentration on noises and because of that a bad focus on passing performance*,” and to lead to (internal) body-focused attention. Furthermore, the feeling of increased focus through impaired hearing was expressed. This, however, at the cost of feeling “*slower*,” “*rather annoyed*” and subjectively perceiving multimodal environmental cues with a delay.

Third, both stadium noise conditions (i.e., positive and negative stadium noise conditions) were stated to impede perception of acoustic Footbonaut sounds and thus, likely increase perceived task complexity, compared to the ‘baseline’ condition (i.e., nine and eight players mentioned this for the conditions, respectively). However, both auditory cue conditions were rated to increase players’ “*focus*” and “*concentration*” to perform in emotionally engaging football practice environments. This notion may be highlighted by one participant’s quote concerning the positive auditory cue condition: “*Because it is very loud, I had to focus more on orientating myse*lf.” Finally, while both stadium noise conditions supposedly led to increased stimulation and motivation (e.g., a player stating: “*the whistling has motivated me*”), the lead researcher’s informal conversations with players after concluding the experiment found that the ‘positive auditory cue’ condition appeared to be slightly more favorable. This condition was explicitly stated to positively influence perceived performance, and perceived positive emotional engagement with the training space. This overall insight may be appreciated by one player’s written quote: “*The singing fans were positively perceived – I found the chanting wicked!*”

## Discussion

While the initial study by Otte et al. (2021) assessed the effect of auditory cue conditions with varying representativeness on football players’ objective passing performances, this article aims at approaching the study from an athlete-centered perspective on individuals’ performance perceptions and emotional engagement with practice under varying auditory noise conditions. Findings are discussed regarding (1) *skilled players’ subjective perception of performance* (i.e., as compared to objective performance data); and (2) *skilled players’ emotional engagement during practice under auditory noise conditions*.

### Skilled players’ subjective perception of performances

The evaluation of players’ perceptions of their own performances compared to actual performance data reveals a strong correlation. The finding that the majority of skilled players correctly perceive and evaluate their own performances (in absence of any augmented feedback of results) aligns with the literature on expertise in sports. Table 1 also demonstrates that players were highly accurate in discerning whether they were successful or not in their performance, with all but one player being aware of which auditory condition they were most successful in. In contrast, players were less accurate in identifying which condition resulted in the fastest or lowest performance. This outcome might be expected in a sport like football, where players are constantly engaged in the dynamics of successful passes, thus arguably possessing expert knowledge of whether a pass was successful or not.

The correlations themselves show an interesting finding. The emotional engagement of the players was significantly correlated with their respective score in all conditions – except the one condition where players could not hear any outside information. In the baseline condition, outside noise from the Footbonaut informed them about their performance, similarly to the positive and negative emotional categories. That effect is not present in the category where the players do not hear any outside information (i.e., where they wear headphones). The correlations are also stronger in the emotionally charged conditions compared to the baseline condition. One reasoning for that might be that outside noise affects the players as a sort of “catalyst” which charges the player’s motivation or reasoning and with that, their performance. A player that was previously defined only by his technical ability (like in the headphone condition) is now rewarded or ridiculed by their outside noise. A good performance then might be enhanced and increased by the noise in the same way a bad performance may be diminished and decreased by crowd noise.

Overall, experiential knowledge of expert performers, here, supports self-monitoring and intrinsic feedback processes that naturally occur within all athletes (Vereijken and Whiting, 1990; Hadfield, 2005; Greenwood et al., 2012). In other words, “it may be argued that over time, athlete knowledge will be superior to that of the coach in some aspects of performance” (Millar et al., 2017, p. 808). This advanced ability to accurately judge own performances may refer back to an ecological view on players’ attuning to their direct environments and thus, developing adequate *knowledge of* information that effectively supports monitoring of performance (Gibson, 1966, 1979; Fajen et al., 2009). Given this high level of player self-awareness, coaches may need to tailor both coaching approaches and informational constraints (e.g., in forms of augmented feedback and instructions) toward players’ needs (Williams and Hodges, 2005; Chow et al., 2016). Put simply, coaches may rather act as (hands-off) facilitators of the practice environment, should consider players’ knowledge and wealth of experience and leave further exploration and problem solving to the players (Millar et al., 2015; Renshaw et al., 2019).

### Skilled athletes’ emotional engagement during practice under auditory noise conditions

Evidently, inducing auditory stadium noise into football practice had some impact on players’ performances and emotional engagement with the environment. This is supported not only by the statistically significant differences for passing times (see Otte et al., 2021), but also in terms of emotional disposition during various practice conditions. Based on an Ecological Dynamics rationale, a critical challenge for coaches concerns the design of holistic athlete practice experiences that support the search for adaptable movement solutions under emotional constraints present in competitive environments (Davids et al., 2013). While continuous interaction between perception, action, and cognition is commonly considered, the presence and role of emotionally engaging training spaces remains underexplored. To theoretically discuss this matter in relation to the concept of *affective learning design* (Headrick et al., 2015), two points appear to be critical: (1) representative and auditory stadium noise environments seemingly increase emotional engagement with the practice design; and (2) individual players rate various auditory noise environments as differently engaging on an emotional level.

First, it is suggested that emotional engagement supports search for relevant action invitations under game-representative informational constraints (Seifert et al., 2013; Headrick et al., 2015). For example, a player unable to hear and communicate with his teammates due to crowd noise may effectively learn to rely on visual environmental information, while the player will also get perceptually attuned to these stadium noises. In other words: “emotions add context to actions” (Headrick et al., 2015, p. 87). Aligned with Constraints-Led Approach, we argue that the manipulation of task and environmental constraints can be critical for skill learning and increased player motivation; e.g., several players stated that both stadium noise conditions influenced emotional engagement in various ways. Thus, facilitating for players to experience training under stadium noise conditions may display one effective way of replicating environmental constraints experienced in football games (Seifert et al., 2017; Otte et al., 2021). Notably, this notion connects back to the aforementioned home-field advantage and crowd noise as one potential reason for it (Pollard, 2008) and hence, the impact of emotionally-laden auditory/crowd environments on players’ and teams’ perceptions and performances warrants further research in this context.

Second, the results indicate that various players had different perceptions of the auditory conditions. This finding is also supported by the original study, which demonstrated that the conditions impacted passing time scores across various situations (Otte et al., 2021). Players in this study cited the four auditory conditions as influencing both performance and emotional engagement, highlighting a critical notion within a Nonlinear Pedagogy (NLP): the necessity for individualized learning designs tailored to players with different skill levels, personalities, and intrinsic dynamics (Renshaw et al., 2009). In simple terms, there is not one ‘cookie-cutter practice drill’ or ideal practice environment (including its auditory noise environment) that is suitable for every athlete, and coaches must be aware of this.

### Limitations and future research

Due to the articles’ highly specialized and unique sample size, findings in this paper should be carefully considered. The decreased representativeness of the practice task in the Footbonaut compared to an actual football task in a packed stadium is also something that should not be discounted easily. Additionally, we would suggest that in future studies with usage of the Footbonaut, the “no noise” earmuff condition could be replaced as a control condition with a condition using non-emotional/non-representative noise, such as white noise. That way, a user of the Footbonaut would still obtain physical information, and one could discern better the effects from stadium information. Furthermore, collected qualitative statements (complementing standardized performance ratings) were open-ended and thus, varied in depth and scope. However, this was deemed acceptable in order to receive an initial and honest insight into players’ emotional engagement and perceptions of the various practice conditions. Notably, since no player was obliged to make statements about particular feelings, any personal comments regarding emotional engagement and perceived performances may carry increased value and display players’ genuine emotional dispositions. Due to the unique sample in this paper, the data set in its depth is limited to an initial overview, demanding future profound investigation by additional studies.

Finally, and due to abovementioned limitations, this research extension may provide a direction for future research on (i) skilled athletes’ abilities to effectively use self-monitoring and intrinsic feedback sources for movement self-regulation; and (ii) athletes’ emotional engagement with different auditory noise environments in practice. Consequently, it is recommended for future studies to investigate these two areas with larger samples of (skilled) athletes.

## Conclusion

Previous studies have aimed to assess football players’ passing performances under auditory cue conditions, such as stadium noise (Otte et al., 2021). The findings of these studies include slower passing times in some auditory cue conditions, which are now supplemented by novel insight into players’ accurate judgments, self-awareness and self-monitoring of their own performances by the data presented in this paper. Thus, these advanced players may benefit from a hands-off coaching approach that focuses on the coach becoming a facilitator, manipulating (task and environmental) constraints within the practice environment. Additionally, use of emotion-laden and affective learning designs may warrant further attention by both researchers studying the effect of representative practice environments and coaches aiming to co-design practice sessions in accordance with principles from an NLP.

## Data availability statement

The original contributions presented in the study are included in the article, further inquiries can be directed to the corresponding author.

## Ethics statement

The studies involving humans were approved by German Sport University Cologne. The studies were conducted in accordance with the local legislation and institutional requirements. The participants provided their written informed consent to participate in this study.

## Author contributions

SK: Conceptualization, Formal analysis, Methodology, Project administration, Supervision, Writing – original draft, Writing – review & editing. FO: Conceptualization, Investigation, Methodology, Writing – original draft, Writing – review & editing. AB: Methodology, Writing – review & editing. TS: Formal analysis, Writing – review & editing. SM: Conceptualization, Supervision, Writing – review & editing.
